# Characterization of Electrospray Ionization Complexity in Untargeted Metabolomic Studies

**DOI:** 10.1021/acs.analchem.4c00966

**Published:** 2024-06-25

**Authors:** William J. Nash, Judith B. Ngere, Lukas Najdekr, Warwick B. Dunn

**Affiliations:** † School of Biosciences, University of Birmingham, Birmingham, West Midlands B15 2TT, United Kingdom; ‡ Institute of Molecular and Translational Medicine, Palacký University Olomouc, Olomouc 779 00, Czech Republic; § Centre for Metabolomics Research, Department of Biochemistry, Cell and Systems Biology, Institute of Systems, Molecular, and Integrative Biology, University of Liverpool, Liverpool L69 7ZB, United Kingdom

## Abstract

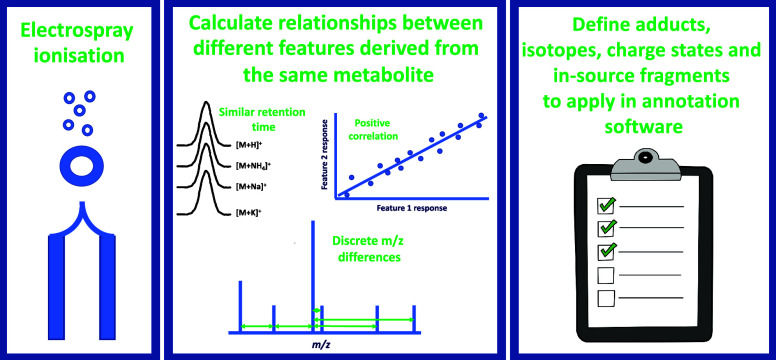

The annotation of metabolites detected in LC-MS-based untargeted metabolomics studies routinely applies accurate *m*/*z* of the intact metabolite (MS1) as well as chromatographic retention time and MS/MS data. Electrospray ionization and transfer of ions through the mass spectrometer can result in the generation of multiple “features” derived from the same metabolite with different *m*/*z* values but the same retention time. The complexity of the different charged and neutral adducts, in-source fragments, and charge states has not been previously and deeply characterized. In this paper, we report the first large-scale characterization using publicly available data sets derived from different research groups, instrument manufacturers, LC assays, sample types, and ion modes. 271 *m*/*z* differences relating to different metabolite feature pairs were reported, and 209 were annotated. The results show a wide range of different features being observed with only a core 32 *m*/*z* differences reported in >50% of the data sets investigated. There were no patterns reporting specific *m*/*z* differences that were observed in relation to ion mode, instrument manufacturer, LC assay type, and mammalian sample type, although some *m*/*z* differences were related to study group (mammal, microbe, plant) and mobile phase composition. The results provide the metabolomics community with recommendations of adducts, in-source fragments, and charge states to apply in metabolite annotation workflows.

## Introduction

Discovery-based untargeted metabolomic studies use a data-driven approach to investigate the metabolite composition of biological samples. The chemical structures of some or normally all of the metabolites (including lipids) are not known prior to data acquisition, and the data collected is applied to derive one or multiple chemical structures for a detected signal. Ultra-high-performance liquid chromatography–mass spectrometry (UHPLC-MS) is the most frequently applied analytical platform in these discovery-based metabolomics studies because of the sensitivity and large number of metabolites that can be detected. The four types of commonly collected data applying UHPLC-MS are the chromatographic retention time (RT), the chromatographic peak area, the mass-to-charge ratio (*m*/*z*) of the intact metabolite (MS1 data), and the fragmentation mass spectrum following gas-phase fragmentation (MS/MS data). Data that are less frequently collected are MS^*n*^ fragmentation mass spectra (where *n* > 2)^1,2^ and ion mobility data.^3,4^

Three types of scientific data (RT, MS1, MS/MS) provide complementary information that is applied to derive one or multiple chemical structures for a detected signal. Chemical structure annotation applying RT and MS/MS data requires collection of reference data for authentic chemical standards that are stored in a library (e.g., see^5^) and are then matched to experimental data collected for biological samples. The size of these databases compared to the proposed size of metabolomes is small and limits the number of metabolite chemical structures that can be annotated applying this strategy.^6^ In-silico prediction of RT^7,8^ and MS/MS mass spectra^9−11^ and molecular networking^12,13^ are areas of significant development to increase the number of metabolites that can be annotated using these types of data. However, annotation of all detected metabolites is not achievable, and it is estimated that only 5–10% of signals are annotated using MS/MS mass spectral libraries.^14^ Therefore, there are many signals for which MS1 data can only be used for annotation without further experimental work.

The metabolite coverage in metabolomic (e.g., HMDB^15^) and chemical (e.g., PubChem^16^) databases is significantly greater compared to RT and MS/MS databases (for example, HMDB v5^15^ contains 220,945 metabolites and a large mass spectral library (mzCloud^17^) contains 12,549 endogenous metabolites (manual and autocurated)). A common approach applied as a first step to annotate metabolites is to use large metabolomic or chemical databases as the starting search space and to use MS1 data to reduce the size of the search space to ideally one, or typically a small number of, molecular formulas (for example, see ref (18)). The reported molecular formulas (and related isomeric metabolites) can then be searched for in RT and MS/MS databases to further reduce the number of possible annotations for a single signal and provide a greater level of confidence for the metabolite’s chemical structure annotation (as defined by proposed reporting standards constructed by the Metabolomics Standards Initiative^19^).

UHPLC-MS platforms predominantly apply electrospray ionization (ESI) to generate charged species (ions) at atmospheric pressure prior to the collection of MS1 and MS/MS data. The ESI source can be described as an electrochemical reactor where both high electrical voltages and high temperatures are applied to both liquid and gas phases containing not only the metabolites of interest but also other chemicals derived from the biological samples (e.g., metals) and liquid chromatography mobile phases (chemical solvents, inorganic/organic salt additives). The combined processes of ionization and desolvation generate multiple ions of different *m*/*z* and the same RT from the same metabolite; these have been defined as metabolite features^20^ or degenerate features.^21^ The complexity of the MS1 data collected is increased because one metabolite can be detected as multiple peaks, and to be able to accurately annotate the metabolite, the ion type needs to be derived to correctly calculate the monoisotopic mass of the metabolite. This complexity was demonstrated in 2009,^20^ and further assessments of the data complexity have been presented more recently.^21^ Adducts (e.g., [M + Na]^+^), isotopic peaks (e.g., ^12^C – ^13^C), in-source fragments (e.g., loss of water through fragmentation in the ion source and mass spectrometer focusing lenses), oligomers, and multiply charged ions are all generated and contribute to this increase in complexity. The first logical step to apply to MS1 data is to group features of the same metabolite together using MS1 *m*/*z* differences, RT similarity, and pairwise response correlation analysis (e.g., see^18,22,23^). Once metabolite features are grouped, determination of the molecular formulas and putative annotation to metabolite chemical structures can be performed.

The current and significant problem in this process is which adducts, isotopes, in-source fragments, and charge states to apply in the metabolite feature grouping process. The application of too few or too large numbers of grouped features from the same metabolite can potentially result in a high proportion of false positives or a low proportion of true positives due to features related to the same metabolite not being grouped together. Best practices and standardization of which adducts, isotopes, in-source fragments, and charge states to apply in the process of metabolite annotation are not presently available in the metabolomics community, and different software apply different lists of adducts and fragments. Therefore, an optimal set of adducts, isotopes, and in-source fragments to use is not known and is not currently applied in the metabolomics community.

As a constructive step to work toward best practices, we report for the first time the characterization of the complexity of ESI-derived metabolite features present in 142 data sets derived from different research groups applying different UHPLC-MS assays and mass spectrometry instruments, which were applied to study different types of biological samples. The data sets have been collected from two metabolomics data repositories (MetaboLights^24^ and Metabolomics Workbench^25^) and the author’s laboratory. We will report the frequency of *m*/*z* differences and relate these to adducts, isotopes, in-source fragments, and charge state. Subsequently, we will investigate whether defined *m*/*z* difference lists could be applied to specific instrument types, sample types, or LC assay types to increase the level of standardization within the metabolomics community.

## Experimental Section

### Sources of Processed Data Sets

In total, 142 data sets (preprocessed *m*/*z*–RT pair intensity matrices) were sourced for this study. All studies with an associated intensity matrix that contained more than nine samples and greater than 999 *m*/*z*–RT features and that were present in the publicly available metabolomics data repositories Metabolights^26^ and Metabolomics Workbench^27^ as of July 1, 2020 were downloaded. These included 36 data sets from 20 studies available in MetaboLights and 73 data sets from 45 studies available in Metabolomics Workbench. A further 33 data sets from 17 studies were provided from the author’s (WD) research group. Within these data, a variety of different mass spectrometers were applied from a range of instrument companies; the same can be said for LC systems and chromatographic columns as well as mobile phases. Sample types throughout are also varied and include mammalian, plant, and microbial samples. The combination of the data sets ensures that the results derived are representative of the multitude of UHPLC-MS configurations and sample types applied in the wider metabolomics community.

### Computational Workflow

The computational workflow applied is visualized in Supporting Information File 1. All data sets were analyzed within the statistical computing software R version 1.3.1056.

#### Step 1: Mass Difference Database Generation (Script 1) (analysis_loop_v2_WN_function_RWall_2020.R)

This function was written by the author (W.J.N.) to calculate the Pearson correlation, *p*-value, and *m*/*z* distance for all possible feature pairs within overlapping RT windows of user-defined width (2 s width and 1 s overlap in this study) in an *m*/*z*–RT intensity matrix for each of 142 data sets separately. The results were stored in an SQL database.

#### Step 2: Database Filtering (Script 2) (sqlwork_20210312_2.R)

Each data set within the SQL database constructed in step 1 was filtered with the filtered results saved into a single new SQL database containing results for all 142 data sets (result.sqlite). All feature pairs with a Pearson correlation coefficient of ≥0.5, *p*-value ≤0.05, and a presence in at least 30% of samples in the data set were retained, and all other feature pairs were deleted.

#### Step 3: Gaussian Kernel Density Estimation (Script 3) (Kernel_Density_Estimation_20210309.R)

For all pairs in the filtered SQL database (result.sqlite), Gaussian Kernel density estimation (GKDE) was performed on all the *m*/*z* distances between the feature pairs from all data sets simultaneously to allow determination of the most common *m*/*z* differences present in the total data. GKDE was carried out using the density() function (bandwidth = 0.0001, N = 2^22, kernel = “Gaussian”, na.rm = TRUE) that is part of the base stats package of R. The *m*/*z* differences were rounded to four decimal places before GKDE was performed. The density and associated *m*/*z* differences were then sorted by density in descending order. Any *m*/*z* difference with a density of less than 0.1 was removed from the list.

#### Step 4: *m*/*z* Difference Counting and Grouping Part 1 (Script 4) (Count_grouping.R)

A grouping process was then carried out using the results from steps 2 and 3. The *m*/*z* distances from each individual data set generated in step 2 were accessed sequentially. The frequency of each four decimal place *m*/*z* difference in each individual data set was counted, and the resulting table was saved in a new SQL database. The GKDE result produced from step 3 was then utilized. The densities and associated four decimal place *m*/*z* differences were sorted into descending density order. The count table for each data set was then grouped using the top *m*/*z* difference density result from the total GKDE data. The top *m*/*z* differences were searched for using a ±0.0005 *m*/*z* window. Upon counting and summing, the *m*/*z* differences were removed from the count table to ensure each *m*/*z* difference is counted once. All rows within the GKDE result table that were within the searched window were removed to avoid spurious count results. Each of the 142 data sets produced a grouped result stored in the data set_count_tables.sqlite.

#### Step 5: *m*/*z* Difference Counting and Grouping Part 2 (Script 5)

A second stage of grouping and summing was performed using the same method as described during the first grouping stage (step 4) using the GKDE result. The input, however, was the already grouped count tables present in the SQL database created during step 4 (data set_count_tables.sqlite). The window applied for grouping was ±0.001 *m*/*z*. The result for each data set was saved in a new SQL database (data set_count_tables_density_merge.sqlite).

#### Step 6: Grouping and Summing GKDE Result (Script 6) (Group_density.R)

The GKDE result was grouped using a window of ±0.001 *m*/*z* using a modified version of the method applied in scripts 4 and 5. Grouped densities were summed together. The result was saved as a.csv file.

#### Step 7: Frequency Table (Script 7) (Frequency_Table_Merge.R)

The grouped GKDE result was used as a reference to the count data from each individual data set to allow merging based on the *m*/*z* differences and the subsequent creation of a frequency table. The SQL database produced during step 5 was used to provide the count data. The result was exported as a.csv file.

### Annotation of *m*/*z* Differences

Annotation of *m*/*z* differences was performed in three stages. The first stage searched for known *m*/*z* differences related to isotopes and charged adducts carrying single or multiple charges and was performed manually by the authors (W.J.N. and W.B.D.). The second stage manually searched for *m*/*z* differences related to known neutral adducts and in-source fragments and separately for metabolic transformations listed in KEGG.^28^ The third stage applied ChemCalc^29^ to convert the *m*/*z* of unannotated *m*/*z* differences to molecular formula(e) using a 10 ppm mass accuracy and the following range of elements: C0–100, H0–100, N0–10, O0–10, S0–10, and P0–10. The fourth stage manually searched for multiply charged differences and differences between already annotated *m*/*z* differences.

## Results and Discussion

### Summary of Data Sets Applied

All publicly available data sets for which intensity matrices after raw data processing were available as of July 1, 2020 and contained *m*/*z*, RT, and peak area data for greater than nine samples and 999 *m*/*z*–RT features were applied in this study. In total, 142 data sets from 82 different metabolomic studies were applied. 61 and 48 data sets were downloaded from the Metabolomics Workbench and MetaboLights data repositories, respectively (65 independent biological studies), noting that many deposited studies did not contain a post-processing data matrix. Thirty-three data sets from the authors laboratory were also included (17 independent biological studies) to increase the diversity of sample types. Data for positive (78 data sets) and negative (64 data sets) ion modes were applied. Primarily, two different chromatography assays were studied, HILIC and normal phase assays (36 data sets) and reversed phase assays (106 data sets). Microbial (10 data sets), plant (26 data sets), and mammalian (102 data sets) sample types were all present and originated from 27 different research institutions. Supplementary file 2 lists information on the data sets applied and provides a summary of the different sample types, ion modes, and assay types.

### Summary and Limitations of Grouping Process Applied

Our approach to identify *m*/*z* differences and their frequency in each data set applied known and routinely used logical rules within data to group metabolite features related to the same metabolite. Here, we applied RT similarity and pairwise peak area correlation analysis to identify *m*/*z*–RT pairs of metabolite features that derive from the same metabolite; specifically, we applied the following criteria to each of the 142 data sets independently in step 1 of the workflow (1) RT difference <2 s, (2) Pearson correlation coefficient >+0.50, and (3) correlation coefficient *p*-value <0.05. GKDE was applied to a *m*/*z* difference list created by integration of data from all 142 data sets. Two hundred seventy-one high-confidence *m*/*z* differences were observed when applying a grouping of 0.001 (related to *m*/*z*) and a density >0.10 (related to frequency of detection); a grouping of 0.001 was chosen after a manual assessment of the GKDE results, which showed that this grouping value would provide grouping of all data around high-frequency peaks. 209 of these were manually annotated by the authors. Supplementary File 3 lists the *m*/*z* differences, putative adduct, isotope, in-source fragment and charge state annotations, and the calculated density for each *m*/*z* difference (the higher the density, the more times it was detected across all studies). The ability to quantify the number of [M + H]^+^ and [M – H]^−^ features was not possible with the approach applied here because the *m*/*z* differences for adducts apply these two ion types as the center *m*/*z* from which *m*/*z* differences can be calculated. It is expected that these two metabolite features would be detected in all data sets. The reporting of oligomers was also not possible using our workflow, and these features will add extra complexity into data sets (for example, see ref (21)). Finally, we applied a 2 s RT window because on plotting raw data for some studies, we observed RT differences of greater than 1 s for the same common adducts of a single metabolite, although this was observed at a low frequency. Applying a smaller RT window is possible and would impact the frequency of some reported *m*/*z* differences. A wide range of adducts, isotopes, in-source fragments, charge states, and biological transformations were annotated. The high number of *m*/*z* differences and the range of annotations (adducts, isotopes, in-source fragments, charge state) demonstrate the high level of complexity in the different types of metabolite features that can be created during and after ESI, observed in metabolomic data sets, and annotated based on the applied workflow.

### Assessment of the Top 20 Ranked *m*/*z* Differences Based on Their Frequency of Detection

In assessing the top 20 most frequently detected and annotated *m*/*z* differences (Table 1), seven were related to *m*/*z* differences between isotopes and included the ^13^C – ^12^C isotopic differences observed as a single (rank 1), double (rank 4), and triple (rank 13) charged ions. The presence of doubly and triply charged ions was not unexpected, although the high frequency in which they are detected is somewhat unexpected and demonstrates that multiple charged ions are frequently formed during ESI. Sodium formate as a neutral non-covalent adduct was the second most frequently detected *m*/*z* difference, and the disodium formate non-covalent adduct was the ninth highest-ranked detected *m*/*z* difference and demonstrates that noncharged salts can non-covalently bind to metabolites in the electrospray process, and this is a more frequent process than the authors expected. Charged adducts and neutral adducts are applied in this manuscript to differentiate between (1) those adducts that directly introduce a charge to the metabolite to form a charged ion (charged adduct, e.g., [M + H]^+^ and [M – Cl]^−^) and (2) those adducts that do not introduce a charge directly to the metabolite or neutralize a charge on a metabolite (neutral adduct, e.g., [M + H + Na formate]^+^). Of importance to note is that many of the studies did not use sodium formate in the mobile phase, and so the source of sodium formate in these studies was not directly related to the mobile phase composition; however, formic acid was applied in the mobile phases and the source of sodium is expected to be derived from the biological samples and/or glassware used in sample preparation and mobile phase reservoirs.

**Table 1:** Twenty Most Frequently Detected *m/z* Differences Observed across 142 Datasets

**rank**	*m*/*z***difference (experimental)**	*m*/*z***difference (theoretical)**	**annotation**	**charge**	**annotation class**	**density**
1	1.0033	1.0034	carbon (^12^C and ^13^C)	1	^13^C – ^12^C isotopic *m*/*z* differences	48.07
2	67.9874	67.9874	CHO_2_Na (sodium for formate)	1	neutral adduct	17.26
3	1.9971	1.9971	chlorine (^37^Cl and ^35^Cl)	1	isotopic *m*/*z* difference—same element	16.85
4	0.5016	0.5017	carbon (^12^C and ^13^C)	2	^13^C – ^12^C isotopic *m*/*z* differences	15.56
5	21.9819	21.9819	M+H]^+^ – [M + Na]^+^ difference	1	*m*/*z* difference between two adducts	13.89
6	0.0001		not annotated		not annotate	10.98
7	18.0106	18.0106	H_2_0 (water)	1	in-source fragment and/or biotransformation	10.45
8	44.0263	44.0262	C_2_H_4_O	1	in-source fragment and/or biotransformation	9.58
9	135.9749	135.9748	CHO_2_Na + CHO_2_Na (sodium formate + sodium formate)	1	neutral adduct	8.92
10	1.0021		not annotated		not annotated	8.45
11	14.0156	14.0157	CH_2_ OR acetate-formate difference	1	in-source fragment and/or biotransformation	8.07
12	2.0157	2.0157	2H	1	in-source fragment and/or biotransformation	7.95
13	0.3343	0.3345	carbon (^12^C and ^13^C)	3	^13^C – ^12^C isotopic *m*/*z* differences	6.70
14	26.0156	26.0157	C_2_H_2_	1	in-source fragment and/or biotransformation	6.49
15	1.0009	1.0009 (Li)	lithium (^7^Li – ^6^Li difference)	1	isotopic *m*/*z* difference—same element	6.40
16	57.9586	57.9586	Na^35^Cl	1	neutral adduct	6.04
17	46.0055	46.0055	CH_2_O_2_ (formic acid)	1	neutral adduct	5.89
18	15.9740	15.9739	[M + Na]^+^ – [M+ ^39^K]^+^ difference	1	*m*/*z* difference between two adducts	5.83
19	28.0312	28.0313	C_2_H_4_	1	in-source fragment and/or biotransformation	5.33
20	2.0062	2.0067	carbon (^13^C + ^13^C − ^12^C + ^12^C difference)	1	^13^C – ^12^C isotopic *m*/*z* differences	5.26

The ^35^Cl – ^37^Cl isotopic *m*/*z* difference was the third most frequently detected *m*/*z* difference. Chlorine can be present as a Cl^–^ adduct in negative ion mode or can be present as a metal salt (e.g., NaCl or KCl) as a neutral adduct in negative or positive ion mode. This observation suggests that chlorine-containing charged and neutral adducts are being observed frequently because a high proportion of metabolites in databases do not contain chlorine in their molecular formula, and so this *m*/*z* difference cannot be primarily related to the presence of chlorine in metabolites (for example, in HMDB, only 1.1% of metabolites contain chlorine). The *m*/*z* difference of 21.9819 was the fifth highest-ranked density, and in positive ion mode, this suggests that this is related to the [M + Na]^+^ adduct (e.g., [M + Na]^+^ – [M + H]^+^*m*/*z* difference). Rank 18 suggests that potassium ions are frequently detected as [M + K]^+^ in the positive ion mode. Rank 15 is defined as the *m*/*z* difference between ^6^Lithium (atomic mass = 6.0151) and ^7^Lithium (atomic mass = 7.0160). Although the presence of lithium in biological matrices and mobile phases is unexpected, some assays do apply lithium salts in lipidomics assays.

Seven of the top 20 *m*/*z* differences were annotated as either biological transformations and/or in-source fragments, and it is not easily possible to differentiate between the two, which complicates the process of converting grouped features into a correct molecular formula. Biological transformations relate to features from two different metabolites and so should not be grouped together in the annotation process, whereas in-source fragments are derived from the same metabolite and so should be grouped together. Six of the seven reported *m*/*z* values could be stable in-source fragments (e.g., water loss (18.0106) and acetaldehyde loss (44.0263)) with the exception of CH_2_ (14.0156), and therefore we propose that their detection could be from both sources, biological transformations and in-source fragmentations. Biological transformations are not a result of ESI but are the *m*/*z* difference between two different metabolites, which might have the same RT resulting in them being positively correlated (including through biological function). Biological transformations were observed across the RT range, although a high frequency was observed at early RTs. At these early RTs, many more metabolites are not retained on the column and co-elute (i.e., many more different metabolites have the same or a very similar RT (± 2 s)) compared to RTs greater than 90 s where fewer metabolites coelute in any defined RT window of ±2 s. Therefore, the probability is higher for two different metabolites to have the same RT and a positive correlation through biological function. These two different metabolites should not be grouped together as two different features of the same metabolite via a biological transformation. However, this can be observed especially for metabolites with a RT related to the void volume and will result in false-positive annotations. To reduce the number of false annotations for metabolites with early RTs, either data is not annotated or biological transformations are not applied for this early RT range. The use of smaller RT windows in the process will not eliminate all possible biological transformations, especially for metabolites with RTs related to the void volume. Two *m*/*z* differences were not annotated, and the *m*/*z* difference of 0.0001 is potentially a result of a small error during data alignment and not a true experimental *m*/*z* difference.

### Assessment of All Ranked *m*/*z* Differences Based on Their Frequency of Detection

Although non-charged (neutral) adducts were expected, the high number of different neutral adducts was surprising at a count of 28 (see Supplementary File 4 for 17 routinely detected neutral adducts).

These included mobile phase solvents (for example, acetonitrile, methanol, and water but not isopropanol), salt-based mobile phase modifiers (for example, formic acid and acetic acid), and other salts including sodium chloride and sodium and potassium formate. Some salts formed multiple adducts including sodium formate, potassium formate, and sodium chloride, and some adducts included multiple forms of the same salt (e.g., sodium formate + sodium formate as a single adduct). Many of these neutral adducts are not listed in the annotation software currently applied. These results highlight that many different molecules present in the sample or introduced during sample preparation (for example, chloroform) and in the mobile phases are not removed from the liquid droplets through the desolvation process of ESI and are therefore retained on charged metabolites as a non-covalent adduct(s). Somewhat surprising was the detection of *m*/*z* differences annotated as HCl (which could be an in-source fragment also) and NaOH. These can either be *m*/*z* differences between two adducts or a neutral adduct depending on ion mode. HCl in negative ion mode is related to differences between two adducts [M – H]^−^ and [M + Cl]^−^ and is not a neutral adduct, but this is not possible in positive ion mode and so must relate to a HCl neutral adduct. NaOH in positive ion mode is related to differences between [M + H]^+^ and [M + Na + H_2_O]^+^ adducts, but this is not possible in negative ion mode and so must relate to a NaOH neutral adduct.^30^

53 *m*/*z* differences were putatively annotated as having a high probability of being in-source fragments as the molecular formula relates to a chemical product expected to be stable in the gas phase. 62 *m*/*z* differences were putatively annotated as biological transformations, and all of these are biological transformations, which are listed in KEGG.^31^ Forty-three of these were also annotated as in-source fragments, which highlights that a *m*/*z* difference can be annotated as two different classes, as discussed in the previous section. Other important results to be noted were that (1) 33 of the *m*/*z* differences were putatively annotated as ions carrying two or more charges, (2) ions containing the following metal atoms were detected—sodium, potassium calcium, copper, iron, zinc, and magnesium, and (3) 62 *m*/*z* differences were not annotated by the authors.

### Are *m*/*z* Differences Consistently Detected as Investigated in 142 Data Sets?

Next, we investigated how frequently each *m*/*z* difference was detected across the 142 data sets. The frequency of *m*/*z* differences for each of the 142 data sets is reported in Supplementary File 5. Subsequently, we removed all *m*/*z* differences that were not annotated and five *m*/*z* differences related to the ^12^C – ^13^C isotopic *m*/*z* difference for charge states one to four because 14 studies contained zero entries for the ^12^C – ^13^C isotopic *m*/*z* difference in charge state one, suggesting that these data sets had been deisotoped. In Supplementary File 5, all frequencies of four or less in columns H to ES were replaced with NA to construct Supplementary Files 6–12 as these were deemed as being infrequently detected within a single data set. 209 *m*/*z* differences and 142 sample sets remained, as detailed in Supplementary File 6.

No *m*/*z* difference was reported in all 142 data sets. The maximum number of studies in which a single *m*/*z* difference was reported was 126 studies, the *m*/*z* difference related to the H–Na pair (noting that this was detected as expected in the positive ion mode but also in negative ion mode). 32 *m*/*z* differences (15.3%) were reported in more than 50% of the studies, and 120 *m*/*z* differences (57.4%) were reported in more than 20% of the studies. This means that 89 *m*/*z* differences (42.6%) were reported in less than 20% of the data sets and 38 *m*/*z* differences (18.2%) were reported in less than 10% of the data sets. Therefore, *m*/*z* differences were predominantly observed in a small number of the data sets studied, and therefore, no single list of *m*/*z* differences would be appropriate to apply in all data sets for metabolite annotation. However, the 32 *m*/*z* differences reported in >50% of data sets would be appropriate to apply in all studies, although study specific additional *m*/*z* difference should be included also.

### Interdata Set Comparison

To further investigate the differences observed, we grouped the studies based on ion mode, UHPLC assay type, MS manufacturer, study group (mammal, microbe, or plant), mobile phase composition, and mammalian sample type (Supplementary Files 7–12, respectively). 183 *m*/*z* differences were reported in five or more negative ion mode data sets, and of these, 182 were observed in five or more positive ion data sets. 202 *m*/*z* differences were reported in five or more positive ion mode data sets, and of these, 182 were observed in five or more negative ion data sets. In summary, the majority of *m*/*z* differences can be detected in both ion modes. This is of relevance because *m*/*z* differences calculated for two charged adducts are thought to be ion mode specific, but the results show that the *m*/*z* difference can be observed in both ion modes and this is important to consider in metabolite annotation workflows.

170 *m*/*z* differences were observed in five or more HILIC/normal phase assay data sets, and all of these were also detected in five or more reversed phase data sets. 203 *m*/*z* differences were observed in five or more reversed-phase assay data sets with 170 of these also being detected in HILIC/normal phase data sets. These results suggest that assay-type-specific *m*/*z* differences are not available for metabolite annotation.

93, 61, 203 and 87 *m*/*z* differences were reported in five or more studies for data sets collected applying Agilent (30 studies), Bruker (11 studies), Thermo Fisher Scientific (67 studies), and Waters (49 studies). The ^63^Cu^37^Cl – ^65^Cu^35^Cl *m*/*z* difference was observed only for Agilent instruments (with the exception of one occurrence for Thermo Fisher Scientific instruments). There were 18 *m*/*z* differences detected by Thermo Fisher Scientific instruments, but not Waters instruments. Only 45 *m*/*z* differences were observed in five or more studies for all four manufacturers. For one MS manufacturer only (Thermo Fisher Scientific), 80 *m*/*z* differences were detected in five or more data sets but in four or less other manufacturers' data sets. These observations suggest that some *m*/*z* differences are instrument manufacturer-specific but many are observed across different manufacturers' instruments.

204, 33, and 104 *m*/*z* differences were reported in five or more studies for data sets studying mammals (104 studies), microbes (10 studies), and plants (26 studies). 126, 82, and 89 *m*/*z* differences were reported in greater than 20% of all studies for mammals, microbes, and plants, respectively. Interestingly, for those *m*/*z* differences reported in greater than 20% of mammal studies (126), 33 were reported in zero or one microbe study, 15 were reported in zero or one plant study, and 11 were reported in zero or one microbe and plant study. These observations suggest that a small number of *m*/*z* differences are study group-specific but many *m*/*z* differences are observed across all study groups.

Mobile phase composition was available for 112 of the studies investigated, and the studies were separated into four groups based on mobile phase similarity. 155 and 123 *m*/*z* differences (of a total of 209) were reported in greater than 20% of studies for two classes ((1) acetonitrile/isopropanol/water (+ salt or acid modifiers) and (2) acetonitrile/water/ammonium formate and/or formic acid, respectively). However, 174 and 143 *m*/*z* differences were not reported in >20% of studies for the other two classes ((1) acetonitrile/water/ammonium acetate and/or acetic acid and (2) methanol/water/formic acid, respectively). Therefore, some mobile phases lead to more *m*/*z* differences being reported than for other mobile phases. However, there is no clear logic to define why this is being observed, acetonitrile and water are present in three of four groups and salts and acids are present in all four groups; no unique solvent or salt or acid is present in only two of the four groups.

Mammalian sample types (blood/serum/plasma, tissue/cell, urine) were also investigated for 88 studies. There was no pattern of *m*/*z* differences being reported for only one or two sample types, and *m*/*z* differences not frequently reported for one sample type were also not frequently reported for the other sample types. Mammalian sample type does not influence the types and frequency of the *m*/*z* differences reported.

### Recommended Use of Isotopes, Adducts, In-Source Fragments, and Charge States

From the results described above, there are lists of isotopes, adducts, in-source fragments, and charge states that can be applied in the metabolite annotation workflow, as derived from 142 data sets collected across the metabolomics community globally. Table 2 lists each class and recommended entries.

**Table 2:** Recommended Charged Adducts, In-Source Fragments, Isotope Pairs, Charge State, and Neutral Adducts to Be Applied in Metabolite Annotation Workflows Applying Electrospray Mass Spectrometry Instrumentation

**charged adducts**	**in-source fragments**	**neutral adducts**
[M – H]	C_2_H_2_	acetic acid
[M + ^35^Cl]^−^	C_2_H_2_O_2_	acetonitrile
[M + ^37^Cl]^−^	C_2_H_4_	acetonitrile + water
[M + H]^+^	C_2_H_4_O	ammonium chloride
[M + NH_4_]^+^	C_2_H_5_NO	calcium formate
[M + Na]^+^	C_2_H_6_	chloroform
[M + ^39^K]^+^	C_2_H_6_O	formic acid
[M + ^41^K]^+^	C_3_H_4_	iron formate
	C_3_H_4_O_2_	methanol
**Isotopes**	C_3_H_6_	potassium acetate
hydrogen (^1^H and ^2^H)	C_3_H_6_O	potassium chloride
lithium (^6^Li and ^7^Li)	C_3_H_6_O_2_	potassium formate
carbon (^12^C and ^13^C)	C_4_H_8_	potassium formate + potassium formate
carbon (^12^C + ^12^C and ^13^C + ^13^C)	C_4_H_8_O_2_	sodium acetate
nitrogen (^14^N and ^15^N)	C_6_H_10_O_5_	sodium chloride
oxygen (^16^O and ^18^O)	C_6_H_12_O_3_	sodium chloride + sodium chloride
magnesium (^24^Mg and ^26^Mg)	CH_2_O_2_S	sodium chloride + sodium formate
potassium (^39^K and ^41^K)	CH_2_O_3_	sodium formate
sulfur (^32^S and ^34^S)	CH_3_N	sodium formate + sodium
chlorine (^35^Cl and ^37^Cl)	CH_4_	sodium formate + sodium formate
chlorine (^35^Cl + ^35^Cl and ^37^Cl + ^37^Cl)	CH_4_O_2_	sodium formate + sodium formate + sodium formate
chlorine (^35^Cl + ^35^Cl + ^35^Cl and ^37^Cl + ^37^Cl + ^37^Cl)	CH_5_N_2_P	sodium formate + sodium formate + sodium formate + sodium formate
iron (^54^Fe and ^56^Fe)	CHNO	sodium hydroxide
copper (^63^Cu and ^65^Cu)	CO	water
zinc (^64^Zn and ^66^Zn)	CO_2_	water + water
zinc (^64^Zn and ^68^Zn)	H_2_	
	H_2_ + H_2_	
**Charge state**	H_2_O + CO_2_	
1	H_2_O_2_P	
2	H_4_O_4_P_2_	
3	NH_3_	
4	NH_3_ + H_2_O	
5	O_2_	

We also recommend the use of 15 isotope pairs related to 10 elements. Of these, all are recommended for use independent of the ion mode and LC assay type, as all are detected frequently in all possibilities. All should be applied for all four MS manufacturers except (1) chlorine (^37^Cl + ^37^Cl + ^37^Cl and ^35^Cl + ^35^Cl + ^35^Cl) and (2) calcium (^40^Ca and ^44^Ca), which was only routinely detected on Thermo Fisher Scientific mass spectrometers, and (3) ^63^Cu^37^Cl – ^65^Cu^35^Cl *m*/*z* difference, which was only detected for Agilent instruments. Eight charged adducts are recommended, five in positive ion mode and three in negative ion mode, and these should only be applied in one of the two ion modes only. We recommend that [M + H]^+^, [M + NH_4_]^+^, [M + Na]^+^, [M+^39^K]^+^, and [M+^41^K]^+^ are applied in positive ion mode only and [M – H]^−^, [M + ^35^Cl]^−^, and [M + ^37^Cl]^−^ are applied in negative ion mode only. Twenty-three neutral adducts are recommended and should be applied across all ion modes, LC assay and MS manufacturer. Thirty-seven in-source fragments are recommended, and of these, all should be applied except two for both ion modes (NH_3_ + H_2_O and C_2_H_5_NO should only be applied in positive ion mode), all should be applied for both LC assay types, and a subset should be applied for each MS manufacturer (it is recommended that only those reported in five or more studies should be applied). Sixty-three biological transformations are recommended. All are recommended for use in both ion modes with the exception of three (H, C_2_H_5_NO, and CH_3_N), all are recommended for use in both LC assays types except five (H, H_2_O_2_P, CH_3_N, C – HN difference, and CH_3_N – O difference), and a subset should be applied for each MS manufacturer (it is recommended that only those reported in five or more studies should be applied). Importantly, biological transformations are not related to two features of the same metabolite, and so this information should be used to ensure two different metabolites are not annotated as two features of the same metabolite. Charge states 1–5 are recommended, and their detection should apply the *m*/*z* differences related to the ^13^C–^12^C isotope pair.

## Conclusions

The data analysis presented characterizes for the first time the complexity of ESI-derived metabolomic data sets collected in laboratories globally using different LC and MS instruments, different sample types, and different LC assays. Complexity is observed in two different ways: (1) more than 200 annotated *m*/*z* differences related to adducts, isotopes, in-source fragments were observed demonstrating the large number of different ion types/metabolite features detected, and (2) no overall logical pattern of the *m*/*z* differences was observed related to sample type, ion mode, LC assay, MS instrument, or research organization. Some *m*/*z* differences not applied in metabolite annotation software have been reported for the first time, including neutral HCl and NaOH adducts.

Although a large number of unique *m*/*z* differences were observed, a much smaller number of *m*/*z* differences were reported for each individual data set. Therefore, the use of large adduct/isotope/in-source fragmentation lists is not advisable, as this is expected to increase the number of false-positive annotations although not necessarily decreasing the number of true positives (the list of possible metabolite annotations will increase in size, but the correct annotation will still be present). It is recommended that the adduct/isotope/in-source fragmentation lists are derived for each data set by using a pre-analysis of the data set prior to metabolite annotation and applying the same strategy as was applied to identify and annotate *m*/*z* differences in the data analysis presented in this paper.

## Data Availability

One hundred and nine data sets are publicly available in either the MetaboLights or Metabolomics Workbench data repositories. Data sets collected in the group of Warwick Dunn are available upon request to the corresponding author.

## Supporting Information Available

(Supplementary file 1) Workflow schematic describing the computational workflow applied to define routinely detected *m*/*z* differences and their detected frequencies including input data, script function, and output data; (Supplementary file 4) list of 17 neutral adducts reported across 142 data sets including single and multiple adducts formed from inorganic salts as well as mobile phase components (PDF)
(Supplementary file 2) information on the data sets applied and a summary of the different sample types, ion modes, and assay types; (Supplementary file 3) *m*/*z* differences, putative adduct, isotope, in-source fragment, charge state annotations, and the calculated density for each *m*/*z* difference reported; (Supplementary file 5) frequency of *m*/*z* differences reported for each of the 142 data sets studied; (Supplementary file 6) detection rate of each annotated *m*/*z* difference for each of 142 studies investigated; (Supplementary file 7) number of studies each annotated *m*/*z* difference was detected in positive and negative ion modes; (Supplementary file 8) number of studies each annotated *m*/*z* difference was detected in HILIC/normal phase and reversed-phase assays; (Supplementary file 9) number of studies each annotated *m*/*z* difference was detected in for different mass spectrometer manufacturer’s instrument; (Supplementary file 10) number of studies each annotated *m*/*z* difference was detected in for different study groups (mammals, microbes, and plants); (Supplementary file 11) number of studies each annotated *m*/*z* difference was detected in for four different mobile phase classes; and (Supplementary file 12) number of studies each annotated *m*/*z* difference was detected in for three mammalian sample types (blood/serum/plasma, tissue and cell, urine) (XLSX)
