# Supplementary material for: Characterization of Electrospray Ionization Complexity in Untargeted Metabolomic Studies
Source: Anal Chem. 2024 Jun 25;96(27):10935–42. doi: 10.1021/acs.analchem.4c00966 (PMC11238156; doi:10.1021/acs.analchem.4c00966)
Supplement: Supplementary file 2 — ac4c00966_si_002.pdf [file ac4c00966_si_002.pdf]

Characterisation of electrospray ionisation complexity in untargeted metabolomic studies

William J. Nash[1], Judith B. Ngere[1], Lukas Najdekr[2] and Warwick B. Dunn[1,3 †]

[1] School of Biosciences, University of Birmingham, Birmingham, West Midlands, B15 2TT, UK

[2] Institute of Molecular and Translational Medicine, Palacký University Olomouc, Olomouc, 779 00, Czech Republic

[3] Centre for Metabolomics Research, Department of Biochemistry, Cell and Systems Biology, Institute of Systems, Molecular, and Integrative Biology, University of Liverpool, Liverpool, L69 7ZB, UK

[†] Corresponding author

Supplementary File 1. Workflow schematic describing the computational workflow applied to define routinely detected *m/z* differences and their detected frequencies including input data, script function and output data.

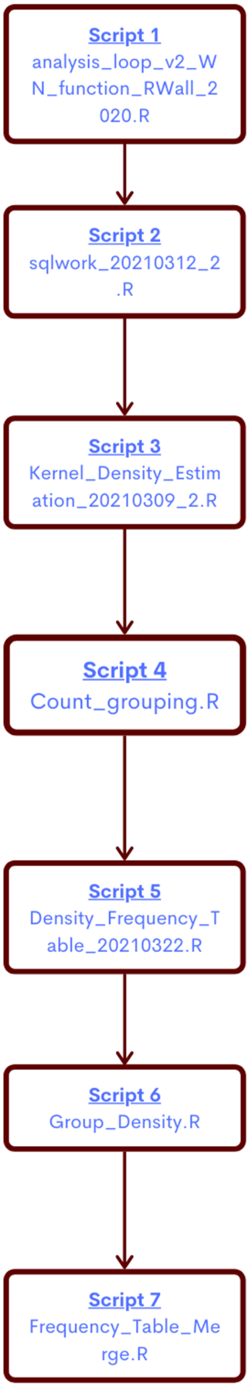

Input 1
A collection of feature intensity matrices containing *m/z* and RT information.
Script function 1
Calculates all *m/z* differences between all feature pairs within 2 second RT windows. Pearson correlation coefficient, *p*-value and n recorded.
Output 1
SQL database containing information on all feature pairs.

Input 2
SQL database output from script 1 containing information on all feature pairs.
Script function 2
Filters feature pairs by Pearson correlation coefficient, *p*-value and n recorded. (R >= 0.5, p <= 0.05, n >= 30% presence).
Output 2
SQL database containing information on all feature pairs meeting the filter criteria.

Input 3
SQL database from script 2 containing all filtered *m/z* distances from each dataset.
Script function 3
Performs Gaussian Kernel Density Estimation (bandwidth = 0.0001, N = 2^22) on all filtered *m/z* distances.
Output 3
CSV containing 4 decimal place *m/z* differences and associated densities across all datasets.

Input 4
GKDE result from all differences produced in script 3 and the list of *m/z* differences from each dataset accessed from SQL database made in script 2.
Script function 4
Counts the frequency of each four decimal place *m/z* difference in each dataset and stores the result. Groups the data and sums frequencies based on the top 4 decimal place values w/ density >= 0.1 from the GKDE results using windows of ±0.0005 *m/z* and stores the result.
Output 4
SQL database containing ungrouped count tables and grouped count tables.

Input 5
SQL database created during script 4 containing grouped count tables and GKDE result from all differences produced in script 3.
Script function 5
Groups the data and sums the frequencies based on the top 4 decimal place values w/ density >= 0.1 from the GKDE results using windows of ±0.001 *m/z*.
Output 5
SQL Database containing secondary grouping step result for each dataset.

Input 6
The GKDE result w/ densities >= 0.1 produced in script 3.
Script function 6
Groups and sums the density data for all four decimal place values w/ density >= 0.1 in descending density order using a window of ±0.001 *m/z*.
Output 6
CSV. containing grouped GKDE result.

Input Data 7
The grouped density data produced in script 6 and the grouped count tables produced in script 5.
Script function 7
Merges the count tables produced in script 6 with the grouped density results produced in script 5.
Output 7
CSV. containing the summed densities of the most common mz differences and the number of times they were counted within each dataset.

Supplementary File 4. List of 17 neutral adducts reported across 142 datasets including single and multiple adducts formed from inorganic salts as well as mobile phase components.

m/z difference (experimental)	*m/z* difference (theoretical)	Annotation	Charge	Annotation class	Density
67.9874	67.9874	CHO_2Na (sodium formate)	1	Neutral adduct	17.26
135.9749	135.9748	CHO_2Na + CHO_2Na (sodium formate + sodium formate)	1	Neutral adduct	8.92
203.9623	203.9622	CHO_2Na + CHO_2Na + CHO_2Na (sodium formate + sodium formate + sodium formate)	1	Neutral adduct	3.04
271.9497	271.9496	CHO_2Na + CHO_2Na + CHO_2Na + CHO_2Na (sodium formate + sodium formate + sodium formate + sodium formate)	1	Neutral adduct	2.50
83.9614	83.9614	CHO_2K (potassium (^{39}K) formate)	1	Neutral adduct	3.77
167.9229	167.9227	CHO_2K + CHO_2K (potassium (^{39}K) formate + potassium (^{39}K) formate)	1	Neutral adduct	1.33
85.9597	85.9595	CHO_2K (potassium (^{41}K) formate)	1	Neutral adduct	0.96
57.9586	57.9586	$Na^{35}Cl$ (sodium chloride)	1	Neutral adduct	6.04
59.9556	59.9557	$Na^{37}Cl$ (sodium chloride)	1	Neutral adduct	3.20
115.9172	115.9172	$Na^{35}Cl$ + $Na^{35}Cl$ (sodium chloride + sodium chloride)	1	Neutral adduct	1.77
18.0106	18.0106	H_20 (water)	1	Neutral adduct and/or in-source fragment and/or biological transformation	10.45
46.0055	46.0055	CH_2O_2 (formic acid)	1	Neutral adduct and/or in-source fragment and/or biological transformation	5.89
41.0266	41.0266	C_2H_3N (acetonitrile)	1	Neutral adduct	2.87
60.0211	60.0210	$C_2H_4O_2$ (acetic acid)	1	Neutral adduct and/or in-source fragment and/or biological transformation	2.45
32.0263	32.0262	CH_4O (methanol)	1	Neutral adduct	1.73
36.0211	36.0211	H_20+H_2O (water)	1	Neutral adduct and/or in-source fragment and/or biological transformation	1.46
117.9142	117.9144	$CHCl_3$ (chloroform)	1	Neutral adduct	1.32
